# Molecular identification and antibacterial properties of an ericoid associated mycorrhizal fungus

**DOI:** 10.1186/s12866-019-1555-y

**Published:** 2019-08-05

**Authors:** O. R. Adeoyo, B. I. Pletschke, J. F. Dames

**Affiliations:** 1 Department of Biochemistry and Microbiology, Rhodes University, Grahamstown, 6140 South Africa; 2 Department of Microbiology, Adekune Ajasin University, Akungba-Akoko, Ondo State P.M.B. 001 Nigeria

**Keywords:** Antibacterial properties, Ericoid mycorrhizal fungus, Molecular identification

## Abstract

**Background:**

The quest for novel sources of antibacterial compounds have necessitated the inclusion of ericoid mycorrhizal fungi (ERM) commonly found within the root of ericaceous plants. Agar-well diffusion method was used to detect antibacterial activity and was followed by the microbroth diffusion method [minimum inhibitory concentration (MIC) and minimum bactericidal concentration (MBC)].

**Results:**

The results of the phytochemical screening indicated that only alkaloids, flavonoids, phenols, saponins, cardiac glycosides and terpenoids were present, while steroids and tannins were absent. The MIC of the extracts ranged between 2 and 16 mg/mL, and the lowest MIC was obtained with *Staphylococcus aureus*. Also, the result of the MBC study indicated that the fungal extract was most active at concentrations of 2 and 4 mg/mL against *Bacillus subtilis* and *S. aureus*, respectively.

**Conclusions:**

This bioassay showed, for the first time, antibacterial activity of *L. incrustata* against some bacterial species. Subsequently, ERM fungi should be given attention when searching for antimicrobial agents because they could provide a solution to solve problems associated with conventional disease treatments (i.e. pathogenic microorganisms resistance).

## Background

Mycorrhizal fungi are members of root endophytes that are capable of complex web interactions with ericaceous plants (e.g., *Erica, Calluna, Rhododendron, Empetrum* and *Rhodothamnus* species). The association that exists between ericaceous plants and mycorrhizal fungi is obligated [1]. This association plays a significant role in determining taxonomy and ensuring the growth of the host plants under harsh conditions [2]. The ericoid mycorrhizal (ERM) association is the most common root-fungus relationship in the ericaceous family [1]. *Leohumicola* species are among the prominent members of ericoid mycorrhizal fungi [3]. They produce two-celled aleurioconidia (single cell conidia formed by projection from the conidiophores) with a spherical to ellipsoidal, dark-brown terminal cells coupled with slightly thickened walls, and cylindrical or cupulate basal cells. They grow slowly on potato dextrose agar (PDA), malt extract agar (MEA) and modified Melin-Norkrans (MMN) media and are naturally found in soil, particularly, at the root of the ericaceous plants [3]. *Leohumicola* is a heat-resistant genus belonging to the hyphomycetes class [4]. The ribosomal small subunit (SSU) and internal transcribed spacer (ITS) sequences of *Leohumicola* genus revealed that the group is different from *Humicola* and *Trichocladium* (Sordariales), and *Thermomyces* (Eurotiales) [4]. Molecular techniques have helped a great deal in unravelling ERM fungal diversity when compared to cultural methods of identification.

Antimicrobial resistance is a significant issue hampering effective treatment of diseases. Scientists are now searching for new potent drugs from novel natural sources, and this will go a long way to support and improve human health. Antibiotic-resistance has been reported with some strains of bacteria such as *Staphylococcus aureus*, *Escherichia coli*, and *Klebsiella pneumoniae*, the report revealed that antibacterial resistance jeopardises the efficient use of drugs in prevention and treatment of different infections caused by microorganisms [5]. *Staphylococcus aureus* is a common causative agent of Staphylococcal infection that combines both nasal carriage and the bacterial immuno-evasive strategies. The illnesses caused may be minor to life-threatening diseases [6].

Secondary metabolites from some fungi have been found to have antiviral, antidiabetic, antitumor, antioxidant, radical scavenging and antibacterial effects [7]. For example, basidiomycetes such as *Fomes fomentarius*, *Boletus edulis, Inonotus obliquus* and *Piptoporus betulinus* have been used to treat gastrointestinal disorders and cancers [7, 8]. Searching for microorganisms with unique and compelling properties is highly desirable because of the burden of contending with diseases (including cancers) that affect human welfare. Phenolic compounds (polyphenols) are secondary metabolites that play a crucial role in supporting human health [9]. Phenolic compounds are made up of an aromatic ring bearing one or more hydroxyl substituent; they range from a simple molecule to complex polymerised compounds [9]. They are found in nature, particularly in foods and microorganism. They are phenolic acids, flavonoids, tannins, hydroxybenzoic (gallic and syringic acids) and hydroxycinnamic acids (caffeic, ferulic, coumaric and sinapic acids) [10]. The present study aims to carry out molecular identification of an ericoid associated mycorrhizal fungus and to determine its antibacterial properties by performing minimum inhibitory concentration (MIC) and minimum bactericidal concentration (MBC) assays. Here, for the first time, we report on an ericoid mycorrhizal isolate showing antimicrobial activity.

## Methods

### Fungal cultivation and production of an antibacterial agent

A fungal isolate (ChemRU330) was used for the investigation. This fungus was obtained from Rhodes University, Grahamstown. Two 5 mm mycelial discs of the actively growing fungus was cultivated in a liquid modified Melin-Norkrans (MMN) medium and was incubated at 28 °C for three weeks, with continuous shaking at 150 rpm on a rotary shaker. After the incubation, the fermentation broth of the fungus was homogenised and filtered through Whatman no. 1 filter paper to obtain a cell-free crude filtrate.

### DNA extraction

Genomic DNA was extracted from pure fungal mycelia using the ZR Fungal/Bacterial DNA Mini-Prep kit (Catalogue # D6005) according to manufacturer’s instructions. DNA concentration was determined by NanoDrop 2000 spectrophotometer (Thermo Scientific, Wilmington, Delaware, USA) and preparations were diluted to make 1–5 ng/mL of DNA template. The integrity of the isolated DNA was evaluated by electrophoresis in a 1% (w/v) agarose gel at 100 V for 75 min in 1X Tris-borate-EDTA (TBE) buffer, stained with 2 μL (concentration 0.5 ng/mL) ethidium bromide and visualised under a Bio-Rad ChemiDoc X-Ray Spectrometer (XRS) system.

### Amplification of the ITS and *Cox1* gene regions

The method described by Nguyen and Seifert (2008) was used, where the ITS region was amplified using the following primers ITS1, ITS4 and ITS5 [11] and KAPA *Taq* ReadyMix (2X). The KAPA *Taq* ReadyMix (2X) is a ready-to-use cocktail containing all components for PCR, except primers and template. The 2X ReadyMix contains KAPA *Taq* DNA Polymerase (1 U per 50 μL reaction), KAPA *Taq* Buffer, dNTPs (0.2 mM of each dNTP at 1X), MgCl_2_ (1.5 mM at 1X) and stabilisers [12]. PCR master mix was made up in a total reaction volume of 50 μl comprising of 25 μL KAPA *Taq* ReadyMix PCR kit (KAPA Biosystems, Catalogue # KK1006), 5 μL template DNA, 2 μL each of both primers (forward and reverse), and 16 μL of water. Amplification was conducted in an automated Applied Biosystems 2720 Thermal Cycler. The cycling parameters used were as follows: initial denaturation at 95 °C for 3 min, 40 cycles at 95 °C for 45 s (denaturing), annealing temperature at 60 °C for 45 s, followed by extension at 72 °C for 1.5 min, and finally 72 °C for 8 min (final elongation). After that, electrophoresis was used as previously described to determine the size of the amplified bands. The *Cox1* gene region was amplified using primers designed for the *Cox1* gene of the Pezizomycotina, PezizF (5′-TCAGGRTTAYTAGGWACAGCATTT-3′) and PezizR (5′-ACCTCAGGRTGYCCGAA GAAT-3′) [3]. The PCR amplification was carried out in a total reaction volume of 25 μL comprising of 12.5 μL KAPA HiFi HotStart ReadyMix PCR kit (KAPA Biosystems, Catalogue # KK2605), 5 μL template DNA, 0.75 μL each of both primers (forward and reverse) and 6 μL of water. An automated Applied Biosystems 2720 Thermal Cycler was used. The initial denaturation temperature was set at 95 °C for 3 min, denaturation temperature at 95 °C for 1 min (40 cycles), annealing temperature 51 °C for 1 min, extension temperature 72 °C for 1 min 30 s, and final elongation temperature 72 °C for 8 min [3]. After that, electrophoresis was used as previously described to determine the size of the amplified bands.

### Sequencing and phylogenetic analysis

After amplification of ITS and *Cox1* barcode regions, the PCR products were cleaned up using a Wizard SV gel, and PCR clean-up kit (Promega, Catalogue # A9281) and the protocol outlined by the manufacturer of the kit was followed. The purified PCR products were sent to Inqaba Biotechnology, Pretoria, South Africa for Sanger sequencing. The sequencing reaction was carried out using the respective primers for IITS region and *Cox1* gene [13, 14]. Nucleotide sequence chromatograms were analysed and edited using Chromas Lite software and compared to sequences in National Centre for Biotechnology Information (NCBI) http://www.ncbi.nlm.nih.gov [15] and UNITE https://unite.ut.ee [16] databases using Basic Local Alignment Search Tool (BLAST) program. Sequences derived from the study and their respective closest matches with homology greater than 95% were pre-aligned in Chromas version 2.6.4 (www.technelysium.com.au) before alignment using BioEdit sequence alignment editor version 6 [17]. To test for phylogenetic relationships, the ChemRU330 sequence and those of species in the genera *Leohumicola* (available in GenBank) were aligned using the ClustalX Version 1.81 [18]. Phylogenetic analysis of *Cox1* barcode region was performed using molecular evolutionary genetics analysis version 7 (MEGA 7) [19]. The evolutionary history was inferred using Neighbor-Joining (NJ) statistical method [20]. The percentage of replicate trees in which the associated taxa clustered together in the bootstrap test (1000 replicates) are shown next to the branches [21]. Bootstrap support values above 50% from 1000 replicates search. The evolutionary distances were computed using Maximum Composite Likelihood method [22] and are in the units of the base number substitutions per site. The analysis involved 25 nucleotide sequences and codon positions included were 1st + 2nd + 3rd + noncoding. All positions containing gaps and missing data were eliminated. There were a total of 628 bp in the final dataset. *Myxotrichum deflexum* was chosen as an outgroup to analysis because of its status as near neighbour to *Leohumicola* clade in the 18S analyses [4].

### Test bacteria

The crude extract of each fungal isolate was screened for antibacterial activity using some bacterial strains as indicator organisms. The indicator bacteria included both Gram-positive (*Bacillus subtilis* and *Staphylococcus aureus*) and Gram-negative (*Escherichia coli*, *Serratia marcescens*, *Proteus vulgaris*, *Shigella sonnei* and *Klebsiella pneumoniae*) bacteria, and were obtained from the undergraduate laboratory of the Department of Biochemistry and Microbiology, Rhodes University, Grahamstown. The bacterial isolates were already identified cultures used for undergraduate practical demonstration. All bacterial cultures were adjusted to 0.5 McFarland standards, which is visually comparable to a microbial suspension of approximately 1.5 × 10^8^ CFU/mL.

### Preliminary screening (using crude fungal filtrate)

The initial screening of antibacterial activity was conducted using a well-dilution method. The nutrient agar (NA) and Luria-Bertani (LB) media were poured into separate Petri plates and inoculated with 50 μL of the bacterial suspension (1.5 × 10^8^ CFU/mL) and spread uniformly by using a sterile glass spreader. Wells (5 mm) were made on the agar media with a sterile cork borer, 50 μL crude filtrate of each of the fungal isolate was placed into each separate well, and the controls [positive (chloramphenicol) and negative (sterile distilled water)]. Parafilm was used to seal the plates before incubation at 37 °C for 24 h. Plates showing antibacterial activity were confirmed by visualisation, followed by measurement of inhibition zones. The average of three repeated trials was taken to evaluate the antibacterial activity [23].

### Qualitative phytochemical screening

Phytochemical screening was conducted on the ethyl acetate extract to check for the presence of the following secondary metabolites – alkaloids, flavonoids, phenols, saponins, steroids, cardiac glycosides, tannins, and terpenoids. Alkaloids: an 80 mg of solid fungal extract was dissolved in 4 mL 2 N HCl. The sample was divided into two portions, one portion was treated with equal amount of Wagner’s reagent, and the second portion was treated with equal amount of Mayers reagent. Reactions showing the appearance of a brown precipitate indicated the presences of alkaloids. Flavonoids (zinc hydrochloride reduction test): to a test-tube containing 1 mL of fungal crude extract, 5–10 drops of dilute HCl, 0.5 g of zinc turnings were added, and the solution was boiled for 2 min. A reddish pink or dirty brown colouration of the solution indicated the presences of flavonoids in the extract. Phenols: a 40 mg crude extract was dissolved in 2 mL of distilled water. Then, a few drops of neutral 5% FeCl_3_ solution was added. A dark green colour indicated the presence of phenolic compounds. Saponins: The crude extract (1 mL) was combined with 5 mL water and shaken for 2 min. The saponins are known to possess frothing activity, the volume of foam was recorded every 10 min. Froth more than 1.5 cm indicated a positive result. Steroids: steroid content was detected by using the Liebermann-Burchard reaction method. A 1 mL fraction of the crude extract was placed in a tube containing acetic anhydride, and a few drops of sulphuric acid (H_2_SO_4_) was added. A bluish-green ring indicated the presence of steroids. Cardiac glycosides: a 1 mL FeCl_3_ reagent (a mixture of 1 volume of 5% FeCl_3_ solution and 99 volumes of glacial acetic acid) was added to 1 mL of the crude extract and was later treated with a few drops of H_2_SO_4_, carefully placed in a dropwise manner along the sides of the test tube. The appearance of greenish blue colour within a few min indicated a positive result. Tannins: the already prepared alcoholic FeCl_3_ reagent was mixed with the crude extract. The mixture produced a bluish-black colour, which disappears on the addition of a few drops of H_2_SO_4_ to yield a yellowish brown precipitate indicates a positive result. Terpenoids: a 1 mL crude extract was added to 1 mL of chloroform, followed by addition of 1 or 2 drops of concentrated H_2_SO_4_ to form a layer. A reddish-brown precipitate at the interface indicated that terpenoids were present [24, 25].

### Extraction and concentration of crude antibacterial compound

The bioactive compound of the fungal filtrate showing inhibition after preliminary screening was then extracted by solvent extraction procedure using ethyl acetate as the organic solvent. To the filtrate, an equal volume of extracting solution (ethyl acetate) was added (ratio 1:1), this was mixed thoroughly for 10 min and kept for 5 min to obtained two clear immiscible layers. The upper tier (bioactive compound) was separated using a separating funnel. The extracting solvent was then evaporated, and the resultant compound was dried in a rotary vacuum evaporator (Buchi Rotavapor® R-200 Rotavapor System) to yield the crude metabolite [26]. The crude extract was then dissolved in distilled water and kept at 4 °C. A known weighed crude extract was dissolved in distilled water before use to obtain a particular concentration.

### Determination of MIC and MBC (cell viability assay using MTT) by microbroth dilution method

The concentrated crude extract was adjusted to a final concentration of 16 mg/mL using a sterile distilled water as the diluent. The test was carried out in duplicate. The MIC was determined using the micro broth dilution method in a 96-well microtiter plate. The test organisms were grown for 24 h at 37 °C. 100 μL of bacterial liquid culture [(optical density adjusted to match 0.5 McFarland standard (1.5 × 10^8^ CFU/mL)], then distributed into a 96-well microtiter plate. The crude extract preparations were diluted to contain different concentrations ranging from 16, 8, 4, 2, 1, 0.5, 0.25, 0.125, 0.0625 and 0.03125 mg/mL dilutions, and were added to the wells containing the bacterial cells. A 5 μg/mL chloramphenicol was used as positive control, and sterile distilled water was used as negative control for the test microorganisms, and the microtiter plates were incubated at 37 °C for 24 h [27]. A 40 μl (0.2 mg/mL) thiazolyl blue tetrazolium bromide [methylthiazolyldiphenyl-tetrazolium bromide (MTT)] was added and at 37 °C for 30 min to detect the MIC. The presence of viable bacterial cells reduced the yellow dye to a pink colour. MIC is the lowest concentration that prevented change and inhibited bacterial growth. MBC was determined by removing a portion of liquid (50 μL) from each well without colour change and placing it on NA, and LB agar then incubated at 37 °C for 24 h. The lowest concentration that yielded no growth after this culturing was regarded as the MBC. All experiments were performed in duplicate [28].

## Results

### Molecular identification and phylogenetic analysis

**Fig. 1:**
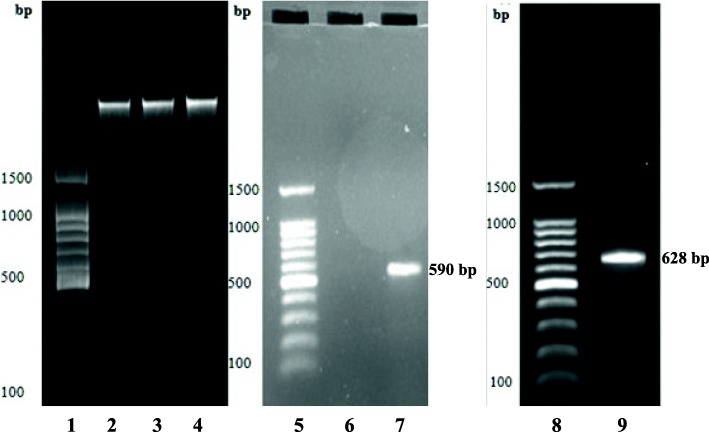
PCR products from DNA extracted from ChemRU330, amplified using primers ITS1 and ITS4 and PezizF-PezizR (*Cox1* gene), stained with ethidium bromide and separated by agarose gel electrophoresis. Lanes 1, 5 and 8 are 100 kb DNA ladder; lanes 2 to 4 are genomic DNA; lane 6 is primer control; lane 7 is amplified ITS sequence; lane 9 is amplified *Cox1* gene sequence

**Fig. 2:**
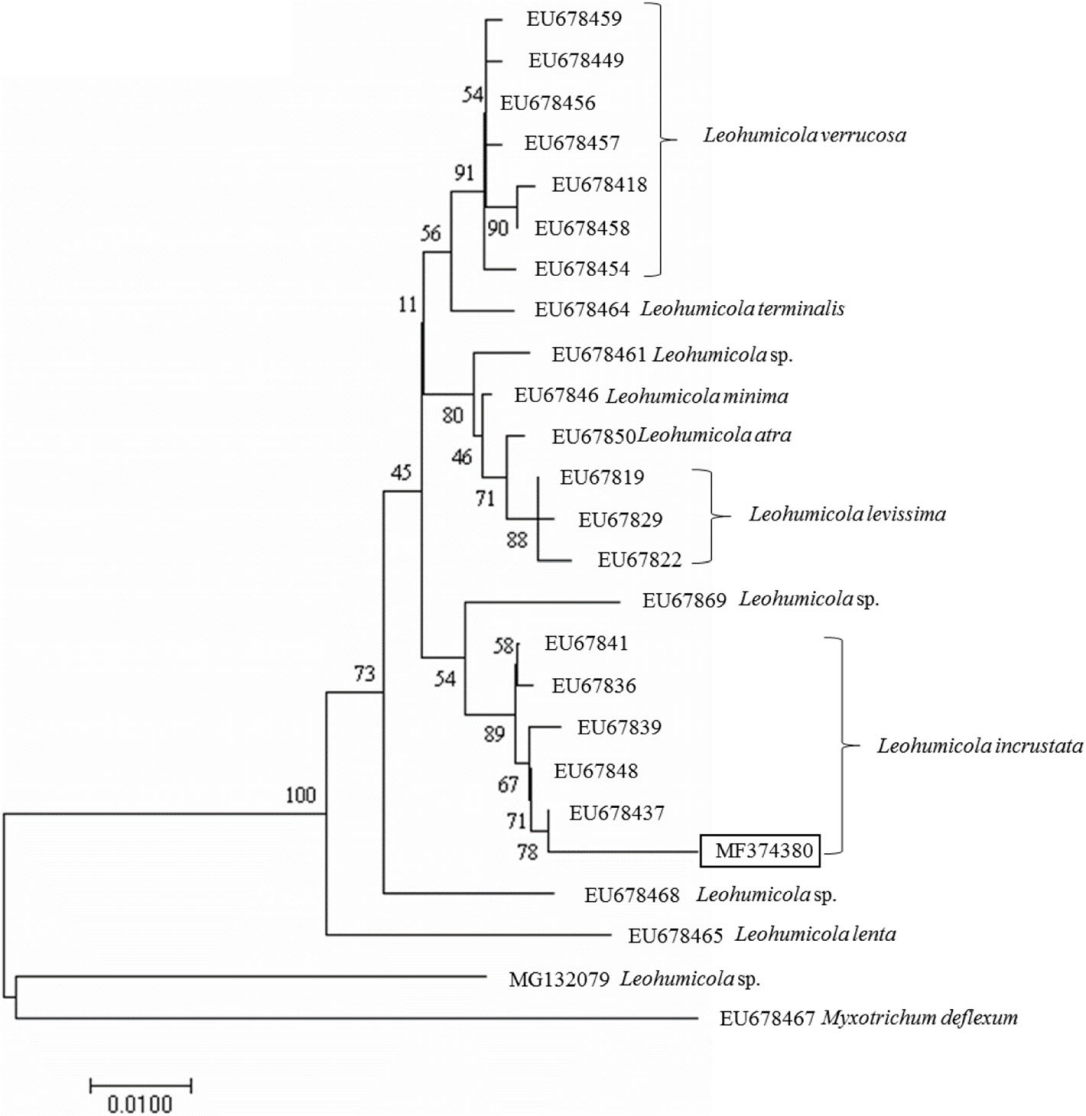
Phylogenetic tree generated from cytochrome oxidase 1 (*Cox1*) gene sequences using the Neighbour-Joining statistical method

This ChemRU330 isolate was previously studied using ITS barcode region (ITS1F and ITS4) (Bizabani, 2015) and was identified as *Leohumicola* sp. Here, *Cox1* gene and ITS barcode regions were used to identify it to species level. The BLAST analysis was sufficient to resolve the organism to a particular name of the ERM fungus. The fungus was determined to have percentage similarities of 99 (GenBank). The sequence of *Cox1* gene matched reference *Leohumicola* sequences in GenBank using BLAST search (Table 1). The closest match to isolate ChemRU330 is with accession number EU678437 and a barcode region of cytochrome oxidase subunit 1 (*Cox1*) of 628 bp (Fig. 1). The internal transcribed spacer sequence was estimated as 590 bp for ITS1-ITS4 (Fig. 1). The ITS sequence read obtained had 497 bp which was adequate to allow taxonomic identification of these fungi to genus level. The maximum composite likelihood analysis of *Cox1* sequences alignment revealed that the *Leohumicola* species form a monophyletic group in a consensus tree (Fig. 2). The bootstrap value of 78% supported the *Cox1* sequence analysis, thus inferred that the isolates (ChemRU330) belong to the *Leohumicola* clade (Fig. 2). The phylogenetic relationships inferred from the ITS region sequences (not shown) did not give sufficient information.

**Table 1:** Identification of some ericaceous plant root associated fungi

Isolate	Primer target region	Genbank accession no.	Closest BLAST match	GenBank accession closest match	Percentage coverage/similarity (GenBank)	Percentage coverage/similarity (UNITE)	Source of closest match
ChemRU330	*Cox1*	MF374380	*Leohumicola incrustata*	EU678437	100/99	ND	South Africa
ChemRU330	ITS	MG209608	*Leohumicola* sp.	KM678361	97/99	99.38	South Africa

*ND* Not detected

### Preliminary antibacterial screening

Table 2 shows the results of initial screening for bioactive compound producing ChemRU330 isolate. In this experiment, crude extract from the isolate was used. The fungus (ChemRU330) inhibited two Gram-positive bacteria (*S. aureus* and *B. subtilis*) and had mild activity against a Gram-negative bacterium, *P. vulgaris.*

**Table 2:** Preliminary screening for antimicrobial activity of *Leohumicola* (ChemRU330) extract against some bacterial species

Sample	*Bacillus subtilis*	*Staphylococcus aureus*	*Escherichia coli*	*Sarratia marcescens*	*Proteus vulgaris*	*Shigella sonnei*	*Klebsiella pneumoniae*
ChemRU330^a^	++	+++	–	–	+	–	–

^a^- = no activity (5 mm); + = slight activity (6–10 mm); ++ = good activity (11–15 mm); +++ = very good activity (< 15 mm)

### Qualitative phytochemical screening

Eight bioactive secondary metabolites that included alkaloids, flavonoids, phenols, saponins, steroids, cardiac glycosides, tannins, and terpenoids were tested for in this experiment (Table 3). The result indicated that only alkaloids, flavonoids, phenols, saponins, cardiac glycosides, and terpenoids were present while steroids and tannins were absent.

**Table 3:** Qualitative determination of the *Leohumicola incrustata* extract for secondary metabolites

S/N	Phytochemical compound	Result
1.	Alkaloids	Positive
2.	Flavonoids	Positive
3.	Phenols	Positive
4.	Saponins	Positive
5.	Steroids	Negative
6.	Cardiac Glycosides	Positive
7.	Tannins	Negative
8.	Terpenoids	Positive

### Determination of MIC and MBC (cell viability assay using MTT)

Table 4 shows that the MIC of the extracts ranged between 2 and 16 mg/mL. *S. aureus* had the lowest MIC (1 mg/mL), followed by *B. subtilis* (2 mg/mL), and *P. vulgaris* (16 mg/mL). The results of the MBC showed that the ChemRU330 extract completely inhibited *B. subtilis* and *S. aureus* at the concentrations of 2 and 4 mg/mL, respectively, while in the case of *P. vulgaris*, there was no growth (MBC = 0). The MBC was confirmed by sampling from wells showing no visible growth as indicated by colour change and when subcultured on NA/LB media.

**Table 4:** The minimum inhibitory concentration (MIC) and minimum bactericidal concentration (MBC) values of *Leohumicola incrustata* extract against four bacterial isolates

Test organism	MIC (mg/mL)	MBC (mg/mL)
*Staphylococcus aureus*	1.0 ± 0.36	4.0 ± 0.31
*Bacillus subtilis*	2.0 ± 0.32	2.0 ± 0.26
*Sarratia marcescens*	0	0
*Proteus vulgaris*	16.0 ± 1.73	0

The values are presented as means ± SEM (standard error of the mean), *n* = 3 per treatment

## Discussion

A sizable number of Ericaceae root-associated fungi have been identified through culture-based techniques and molecular analysis [29]. But, there are still limited reports available on the identity of *Leohumicola* species using sequences generated through the ITS barcode region analysis. Subsequently, the *Cox1* barcode region was adopted to facilitate identification to species level allowing the specific name of the organism to be inferred. The ChemRU330 isolate was inferred to be *L. incrustata*. Similar, Nguyen and Seifert (2008) reported three new species (*L. levissima, L. atra and L. incrustata*) from the United States and South Africa using both the ribosomal internal transcribed spacer (ITS) and cytochrome oxidase 1 (*Cox1*) as DNA barcodes for the identification of *Leohumicola* species. Also, this observation was made by Hambleton co-workers (2005). A large proportion of the ERM fungi and related fungi are now being identified using both GenBank and UNITE databases [4, 30]. It should be noted that UNITE database contains ITS sequences generated from identified fungal sporocarp voucher specimens [16] and are suitable for studying mycorrhizal fungi.

For phylogenetic relationships, the taxonomic status of the ChemRU330 isolate was inferred only from the *Cox1* gene sequence while ITS sequence generated in this study was inadequate for this purpose and was disregarded. Also, the results here showed that NJ statistical method is capable of producing a reasonably accurate parsimonious tree [22].

Some bacteria used were susceptible to the crude extract of *Leohumicola incrustata*, particularly the Gram-positive bacteria. The results obtained are promising and even bacteriostatic for a Gram-negative bacterium (*P. vulgaris*). The presence of some phenolic compounds (phenolic acids and tannins) in the extracts was responsible for the bioactivity. Reports by some authors indicated that phenolic compounds with antibacterial properties are common features of natural matrixes such as microorganisms [27, 28, 31]. Also, we observed that the slightly inhibited *S. marcescens* (Gram-negative bacterium). This agrees with the results reported earlier on *E. coli*, *K. pneumonia* and *P. aeruginosa* [28, 32] who noticed negative activity against these organisms. The screening for phytochemicals revealed the presence various bioactive compounds that include phenolics, alkaloids, flavonoids, cardiac glycosides and terpenoids. The production of phenolic compounds and other bioactive compounds in endophytic fungi [24, 33] and mushrooms [8, 28] have been vastly reported.

Considering the problems associated with currently available antibiotic drugs (antibiotic resistance), there is the need to discover new antimicrobial agents. In this study, the antimicrobial potential of a mycorrhizal fungus (ChemRU330) was investigated to determine the MIC and MBC. The results showed that the fungus contains antibacterial compounds which should be explored to complement the already available drugs for active disease treatment. The MIC reported in this study is low enough and promising for antibiotics production. Table 5 shows that the MIC value obtained against *Staphylococcus aureus* is similar to those reported for *Tremetes gibbosa, Pleurotus ostreatus* and *Amanita citrina* while better MIC values were reported for *Xylaria* sp., *Fusarium* sp., and Oxacillin [8, 31, 34–37]. Further purification and characterization of active compound(s) will increase the effectiveness of the antimicrobial agent. It has been suggested that antimicrobial compounds produced from fungal endophytes are highly rated because they are potent and more stable, probably due to their gene recombination with the host plant tissues [38]. Also, about 80% of endophytic fungi have either antibacterial, fungicidal or herbicidal properties [39]. The progressive exploration of new and novel antimicrobial compounds is principally aimed at overcoming the difficulties associated with resistant pathogens [40].

**Table 5:** The MIC and MBC of crude extracts of *Leohumicola incrustata* and some antimicrobial agents against *Staphylococcus aureus*

Antimicrobial agent	MIC (mg/mL)	MBC (mg/mL)	Reference
*L. incrustata*	1	4	Current study
*Tremetes gibbosa*	6	30	[34]
*Fusarium* sp.	0.016	na	[35]
*Pleurotus ostreatus*	6	na	[31]
*Amanita citrina*	5	5	[8]
*Xylaria* sp*.*	0.004	na	[36]
Oxacillin	0.002	na	[37]

*na* MBC value not available

## Conclusion

Some root endophytic fungi such as the ERM fungi should be extensively studied and considered as additional sources of new antimicrobial agents in drug and food preservation. This bioassay showed for the first time, the antibacterial activity of *L. incrustata* against some bacterial species.

## Data Availability

The fungal isolate used for this study was cultured from roots of ericaceous plants (*Erica chamissonis*) (Bizabani 2015). *Leohumicola incrustata* (Isolate code ChemRU330/Genbank Accession Number MF374380/The South African National Collection of Fungi Accession Number PPRI 17268), was obtained from Mycorrhizal Research Laboratory, Rhodes University, Grahamstown.

## Abbreviations

*Cox1*: Cytochrome oxidase 1
DNA: Deoxyribonucleic acid
EMR: Ericoid mycorrhiza
ITS: Internal transcribed spacer
MBC: Minimum bacteriacidal concentration
MIC: Minimum inhibitory concentration
MMN: Modified Merlin-Norkran
MTT: Methylthiazolyldiphenyl-tetrazolium
PCR: Polymerase chain reaction
